# Characterization of Physico-Chemical Properties and Antioxidant Capacities of Bioactive Honey Produced from Australian Grown *Agastache rugosa* and its Correlation with Colour and Poly-Phenol Content

**DOI:** 10.3390/molecules23010108

**Published:** 2018-01-05

**Authors:** Sushil Anand, Edwin Pang, George Livanos, Nitin Mantri

**Affiliations:** 1 The Pangenomics Group, School of Science, RMIT University, Melbourne 3083, Victoria, Australia; sush.anand@gmail.com (S.A.); eddie.pang@rmit.edu.au (E.P.); 2 Kenkay Pharmaceuticals Pty Ltd., Smeaton Grange 2567, NSW, Australia; george@kenkay.com.au

**Keywords:** honey, antioxidant, DPPH•, ABTS•+, ORAC, FRAP, *Agastache*, manuka, Jelly bush, Australian honey

## Abstract

The antioxidant and antimicrobial components of honey vary based on sourced of nectar. Medicinal plants with the therapeutic value have potential to produce honey with greater bioactivity. The aim of the present study was to characterize the physico-chemical and antioxidant capacities of *Agastache* honey produced from *Agastache rugosa* and compare them with other popular commercial honeys sold in Australia. The total phenolics, total flavonoids, moisture content, colour, pH, protein content and antioxidant capacity were evaluated for *Agastache*, Manuka, Jelly bush, Tea tree, Super manuka and Jarrah honeys. The results reveal that the moisture content ranged from 17–21%, pH ranged from 3.8–4.3 and estimated protein content ranged from 900–2200 µg/g. The DPPH•, ABTS•+, ORAC and FRAP methods were used to measure the antioxidant capacity of the honey samples. The DPPH• % inhibition, ABTS•+, ORAC and FRAP values for *Agastache* honey were 9.85 (±1.98 µmol TE/g), 26.88 (±0.32 µmol TE/g), 19.78 (±1.1 µmol TE/g) and 3.61 (±0.02 µmol TE/g) whereas the highest antioxidant capacity values obtained were 18.69 (±0.9 µmol TE/g), 30.72 (±0.27 µmol TE/g), 26.95 (±0.9 µmol TE/g) and 3.68 (±0.04 µmol TE/g), respectively. There was a positive correlation between colour, total phenolic content and DPPH• scavenging activity for most of the honeys except Tea tree honey. However, there was no clear correlation with ABTS•+, ORAC and FRAP values. The measured antioxidant capacity of samples varied with the assays used. The DPPH• assay clearly indicated that the phenolic compounds contribute to the scavenging activity of the honeys. Nevertheless, all assays confirm that *Agastache* honey has significant antioxidant capacity. Therefore, *Agastache* honey can be important to human nutrition and health.

## 1. Introduction

Honey is a superior functional food which has been utilised for many years in the traditional and complementary medicine as a remedy to the challenges posed by microbial diseases. Recently, its role in the treatment of burns, gastrointestinal disorders, infected and chronic wounds, skin ulcers and eye ailments has been highlighted [1,2]. The efficacy of honey is partially due to its antibacterial property which is chiefly contributed by the accumulation of hydrogen peroxide and other non-peroxide factors such as lysozyme, pH, phenolic acids and flavonoids [3,4].

Stress (physical, chemical or biological) leads to oxidative damage that is considered to be root cause of most diseases. Oxidative stress is responsible for damage to cellular components including the DNA. There are various reports indicating the involvement of oxidative stress in the pathogenesis of various disorders and diseases [5]. Natural antioxidants contained in foods, fruits, beverages and herbs have therefore drawn global attention and are marketed as nutraceuticals and pharmaceutical agents [6,7,8]. In a previous study, it was demonstrated that antioxidant activity of honey on a fresh weight basis is equivalent to that of many fruits and vegetables [9]. Furthermore, honey antioxidants can prevent oxidation reaction in food such as enzymatic browning of fruits and vegetables [10], lipid oxidation in meat [11] and inhibit the growth of food borne pathogens and food spoilage organisms [12]. In humans, the consumption of honey increases the plasma antioxidants which provide protection to the cells in the bloodstream indicating that bioavailability and bioactivity of honey gives a high efficiency antioxidant transfer from honey to plasma [13].

The main honey constituents which contribute to the antioxidant activity are phenolic acids, flavonoids, glucose oxidase, catalase, ascorbic acid, protein and carotenoid [14]. Some authors have studied the phenolic and flavonoids content of honey to establish their correlation with antioxidant activity. Beretta et al. reported significant positive correlations between phenolic concentration, antioxidant capacity and the colour of honey [15]. Bertoncelj et al. studied Slovenian honey and observed a positive correlation between the parameters [16], Socha et al. observed correlation between Total phenolic content and antioxidant activity in Polish honeys [17], Ferreira et al. studied Portuguese honey [18] and Pontis et al. studied Brazilian honey and observed that honeys with darker colour have a higher total phenolic content reflecting higher antioxidant activity [19].

The antioxidant and antimicrobial components of honey are sourced from the plant species. Medicinal plants with the therapeutic value can therefore be exploited to produce honey with greater bioactivity. In the present study, *Agastache rugosa* (*Lamiaceae* family), a medicinal plant was selected to produce mono-floral honey It is a perennial herb ubiquitous in the fields of Korea which has been used as a wild vegetable and a herbal drug (leaves) in traditional therapies [20]. It is used in Chinese traditional medicine for the treatment of cholera, vomiting and miasma and has been reported to have antitumor, antimicrobial, antifungal and cytotoxic activities [21,22]. Sylwia reviewed the bioactivity of *Agastache rugosa* and stated that the medicinal properties of aerial part of *A. rugosa* (essential oil and methanol extracts) are well-established and they have been used in combination with other herbs [23]. The volatile compounds from leaves, flower spikes and nectar of *Agastache* were recently analysed [24]. Additionally, other authors have reported the antioxidant capacity of essential oil from *Agastache* calyx and water extracts of its leaves. The antioxidant activity has been suggested to be due to presence of non-volatile phenolic compound tilianin [25]. The present study was conducted to characterize physico-chemical properties and antioxidant capacities of *Agastache* honey and its correlation with colour and poly-phenol content.

## 2. Results and Discussion

### 2.1. Honey Samples

In general, mono-floral honeys are classified on the basis that the honey must derive at least 51% of the constituent nectar or 45% of contaminant pollen from a single floral source. In this study, however, *Agastache* honey was produced from a single floral source in a closed glass-house. Therefore, it was pristine mono-floral honey. Raw *Agastache* honey (~500 g) was successfully harvested, filtered and stored in the dark at 4 °C. The colour intensity, phenolic content, flavonoid content and antioxidant capacity was characterized for *Agastache* honey produced by us and compared with commercially-important honeys sold in Australia including Manuka honey (hnz, UMF 22+, *Leptospermum scoparium*), Tea tree honey (Miellerie, *Leptospermum lanigerum & Leptospermum scoparium*), Jelly bush honey (Australia’s Manuka, 20+Active, *Leptospermum polygalifolium*), Super manuka honey (Berringa, MGO-400, *Leptospermum polygalifolium*), Jarrah honey (Elixir, TA 45+, *Eucalyptus marginata*).

### 2.2. Physico-Chemical Properties

All the Australian honeys including Manuka were found to be acidic and their pH values ranged from 3.8 to 4.26. The Jarrah honey was least acidic (4.26) as shown in Table 1. These values were within the pH range of 3.81 to 6.32 as reported by Chandler et al. for Australian honey [26]. However, the pH of Manuka honey (4.0) was lower than previously reported (4.4) [27]. The acidity of honey is responsible for its flavour and stability against microbial spoilage. Moreover, honey pH plays a key role in the wound management when applied topically. Chronic non-healing wounds have an elevated alkaline environment and healing occurs more readily in an acid environment [28].

The moisture content (%) in the investigated honey samples ranged from 13–21%. Except Jarrah honey (21%), all other honeys had moisture content below 20%, which is the maximum recommended limit for the moisture content as per the International Honey Commission or Codex standard for honey (Codex, Alimentarius) [29]. The *Agastache* honey we produced had 17% moisture content confirming that good quality mature honey was harvested from the hives. The measured moisture content for Australian honeys was consistent with the previously reported [26]. The moisture content of the honey is influenced by various factors such as harvesting season, degree of maturity reached in the hive and climatic factors [30].

The protein content of the investigated honey samples ranged from 900–2200 µg/g (Table 1). Our *Agastache* honey had considerable amount of protein: 1428 µg/g. When compared to other honey samples, Jarrah honey contained highest amount of protein: 2178 µg/g. This protein content is comparable to that found in Brazilian honeys where it was varied from 199 to 2236 µg/g [31]. The protein content of honey is normally less than 5000 µg/g [32]. The proteins and amino acids concentration is influenced by the floral sources, geographical origin and storage time. The protein content is mainly comprised of enzymes, glyco-proteins and some antimicrobial peptides such as bee-defensine-1, which are either introduced by the bees or derived from the nectar [33].

Colour characteristic was determined by reading absorbance of honey at A_450_. The colour intensity of analysed samples ranged from 461 mAU for the lighter colour (*Agastache* honey) to 1135 mAU for the darker colour (Jelly bush honey). The order of darker to lighter colour honey was as follows: Jelly bush, Manuka, Super manuka, Jarrah, Tea tree honey and *Agastache* honey. The colour intensity of Manuka honey was higher (1007 mAU) than that previously reported (805 mAU) by Moniruzzaman et al. [34]. The colour of honey determines the presence of phenolics, flavonoids and pigments present in them. The previous studies demonstrated that the darker colour honey has a higher amount of antioxidants in the honey [19]. However, it should be noted here that although the commercially obtained Australian honeys tested here are claimed to be mono-floral, technically, they need to contain only more than half nectar from single source species. Therefore, the phenolic content and colour of these honeys could well be influenced by other nectar sources around these species. Comparatively, the honey we produced was in a closed enclosure and therefore was mostly pure *Agastache* honey.

The total phenolic content (TPC) was determined by Folin-Ciocalteu’s method. The mean values and standard errors are shown in the Table 2. The results reveal significant differences between the honeys analysed. According to the results, *Agastache* honey had the lowest TPC value (853.6 ± 5 µg GAE/g of honey) and Jelly bush had the highest TPC value (1415.6 ± 126 µg GAE/g of honey). The TPC of Jelly bush honey was higher than that previously reported (121.1 µg GAE/g of honey) for Australian Tea-tree honey (*M. quinquenervia*) [35]. A study on Portuguese honeys also reported that the amber colour honey had the average values of (406.2 ± 17.2 µg GAE/g) and light-coloured honeys had the average values of (226.1 ± 0.2 µg GAE/g) [14]. In the present study, the TPC values for all the honeys were higher than Portuguese honeys indicating that all honeys had amber colour and lighter coloured honeys such as *Agastache* and Tea-tree had higher TPC value (1263.5 ± 143.1 µg GAE/g). The most common method used to estimate phenolic content of the food sample is Folin-Ciocalteu’s method. However, Shahidi et al. reported that the reagent used in this method also reacts with non-phenolic reducing compounds such as some sugars and amino acids, which may lead to an overestimated absorbance value but this is the most acceptable method [36].

The total flavonoids content (TFC) of different honeys analysed is shown in Table 2. Jelly bush honey contained highest flavonoids content (53.9 ± 10.9 µg CE/g of honey) while Tea-tree honey had lowest flavonoids content (20 ± 4.3 µg CE/g of honey). The TFC values obtained in the analysed samples was significant when compared with other honey TFC values such as Australian Tea-tree honey (63.5 µg QE/g of honey), Australian heath (21.2 µg QE/g of honey), brush box (45.3 µg QE/g of honey) and Australian sunflower (17.9 µg QE/g of honey) [37]. TFC was quantified spectrophotometrically with aluminium chloride which determines flavones and flavonols whereas flavanones are determined by 2,4-dinitrophenylhydrazine method. However, this method is widely used to determine the TFC [38].

### 2.3. Antioxidant Capacity

The antioxidant capacities of *Agastache* honey produced by us and commercially important Australian honeys including Manuka were evaluated. To evaluate antioxidant capacity there are numerous methods available for food and biological samples but there is no standardized method that can measure the antioxidant capacity of all samples accurately. Therefore, antioxidant capacity of honey samples was measured by four common methods, namely, DPPH•, ABTS•+, ORAC and FRAP assays. In order to compare results obtained from different assays, trolox (an analogue of Vitamin E) was utilized in the study as a reference standard and antioxidant capacity was calculated per gram of honey sample and expressed in micromoles trolox equivalent per gram of honey.

The radical scavenging capacity of honey samples was determined by DPPH• assay and TEAC assay. DPPH• assay and TEAC assays are fast, economical, reliable and frequently used in the laboratories. In DPPH• assay, the antioxidant values are usually expressed in % inhibition of scavenging activity of the sample, trolox equivalent EC50 values or sample concentration required to inhibit 50% of the free radical scavenging activity. In this study, a standard calibration curve was constructed (*y* = 0.1603*x* + 14.355, *R*^2^ = 0.9889) to obtain the % inhibition of scavenging activity. For TEAC assay, a calibration curve with equation (*y* = 0.287*x* − 1.719, *R*^2^ = 0.998) was constructed. Usually, a high DPPH• and ABTS•+ scavenging activity relates to high levels of antioxidant activity.

The results obtained for antioxidant capacities in all assays are shown in Table 2. The scavenging activity of free radicals determined by DPPH• assay indicated that the highest antioxidant activity was present in Manuka honey (18.69 ± 0.9 µmol TE/g) followed by Jelly bush, Tea tree, Super manuka, *Agastache* and Jarrah honeys. Similarly, the scavenging activity of free radicals determined by TEAC assay indicated that the highest activity exhibited by Manuka honey (30.72 ± 0.27 µmol TE/g) followed by *Agastache*, Jelly bush, Super Manuka, Jarrah and Tea tree honeys. The antioxidant capacity exhibited by *Agastache* honey in TEAC assay (26.8 ± 0.32 µmol TE/g) was more than twice the capacity shown in DPPH• assay (9.85 ± 1.98 µmol TE/g). The reducing power of antioxidants was determined by FRAP assay. The results were expressed as the concentration of antioxidants having a ferric reducing ability equivalent to trolox. A calibration curve with equation (*y* = 0.0024*x* + 0.0377, *R*^2^ = 0.9307) was obtained for trolox. The values obtained for antioxidant capacity of honey in the FRAP assay was lower than the values obtained for DPPH• and TEAC assay. The highest antioxidant capacity was exhibited by Manuka (3.68 ± 0.04 µmol TE/g) followed by *Agastache*, Jelly bush, Jarrah, Super manuka and Tea tree. The antioxidant capacity of Manuka honey exhibited higher FRAP value than 1.21 as previously reported by Alzahrani et al. [27].

**Figure 1:**
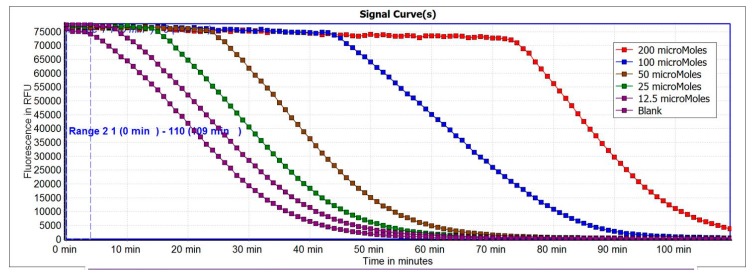
Fluorescence decay curves of different concentrations of Trolox.

Oxygen Radical Absorbance Capacity (ORAC) assay is widely used to measure the antioxidant capacity of foods, nutraceuticals and pharmaceuticals and uses biologically relevant free radicals [39]. In this assay, a calibration curve was obtained by plotting the area under the curve (AUC) against trolox concentrations in the 0–200 µM range (Figure 1). The equation of the calibration curve obtained was *y* = 18844*x* + 359391 with a good R squared value (*R*^2^ = 0.991). The antioxidant values were determined from the standard curve. The antioxidant capacity of honey samples measured by the ORAC assay followed the order: Jelly Bush > Manuka > *Agastache* > Teatree > Super manuka > Jarrah. These capacities were found equivalent with the antioxidant capacities of some fruits and vegetables (in the range of 0–19 µmol) suggesting that honey may be comparable to fruits and vegetables in antioxidant capacities [40,41]. However, the ORAC values reported for honeys by Gorjanovic et al. was in the range of 3.7–12.9 µmol TE/g which was lower than the present findings (a range of 12.4–26.9 µmol TE/g). Another study reported a range of ORAC values 2.00–21.07 µmol TE/g for a variety of honeys containing highest value of strawberry honey [15,42]. Whilst, Brudzynski et al. analysed 20 Canadian honey samples and reported the ORAC values in the range of 1.99–19.77 µmol TE/g with Buckwheat honey obtaining highest ORAC value and Manuka honey obtaining lower ORAC value of 8.19 µmol TE/g than the present finding [43].

Overall, the antioxidant capacities of honeys varied based on assays used. The variable antioxidant capacity obtained in different assays may be attributed to the kinetics of the different reactions. In general, the assays used are based on hydrogen atom transfer (HAT) reaction and single electron transfer (ET) reaction. The ET based assays involve one redox reaction with the oxidant as an indicator of the reaction endpoint. Most HAT based reactions monitor competitive reaction kinetics and the quantitation is derived from the kinetic curves. HAT based assay is Oxygen radical absorbance capacity (ORAC) in which antioxidants and substrates compete with thermally generated per-oxyl radicals. ET based assays measure the capacity of an antioxidant in the reduction of an oxidant which changes colour when reduced [44]. The degree of colour change is correlated with the samples antioxidant concentrations. ET based assay are ABTS•+, DPPH• and FRAP assay.

### 2.4. Correlation between Parameters

A correlation matrix was created to analyse the relationship between different parameters. The parameters that had significant inter-relationships (except Tea-tree honey) are presented in Table 3. A significant positive correlation between colour intensity and TPC was observed (*R* = 0.944, *p* ≤ 0.01, Table 3). There was a strong positive correlation observed between TPC and TFC (*R* = 0.866, *p* ≤ 0.05). Similar findings were reported with correlation coefficient *R* = 0.85 by Al et al. [45] but other authors reported that there is no correlation between these two characteristics [46].

Colour intensity and TPC was found positively correlated with the antioxidant capacity determined by DPPH• assay while TFC was not found significantly correlated (0.484, *p* ≤ 0.40) with DPPH• assay. However, there was no significant correlation observed between colour intensity and TPC with the antioxidant capacities determined by ABTS•+, FRAP and ORAC assays. The studies conducted on Malaysian honey reported the significant correlation between TPC (*R*= 0.965) and TFC (*R* = 0.888) with the DPPH•-scavenging activities [47]. Moreover, the study on Cuban honeys reported correlation between TPC and TFC (*R* = 0.831) also [9]. These data clearly indicated that the antioxidant property of honey samples is dependent on total phenolic content. Additionally, more studies are required to be conducted on a variety of honeys to establish the correlation of colour intensity, TPC, TFC and antioxidant capacity.

Notably, as far as honey colour is concerned, Tea tree honey colour intensity was low and there was no significant correlation observed between colour intensity, TPC and DPPH• assay. However, a significant correlation was observed between TPC and DPPH• assay which implies that the honey colour does not necessarily represent the higher content of phenolic compounds and antioxidant activity in all cases. Moreover, the colour intensity of the honey is related to the presence of pigments (carotenoids, flavonoids etc.) which are known to have antioxidant properties [48]. In the present study, this view is supported by the presence of low content of flavonoid in tea-tree honey indicating, however, that its antioxidant activity is chiefly due to phenolic contents.

Additionally, antioxidant capacity of honey can be attributed to the wide range of compounds such as phenolic compounds, organic acids, small peptides and enzymes [49]. But, there was no statistically significant correlation observed between protein content and DPPH• radical-scavenging activity (*R* = −0.712, *p* = 0.177) indicating that the protein content is not relevant to the antioxidant capacity but it may contribute to the other properties (antimicrobial and anti-inflammatory activity) of the honey.

All methods have advantages and disadvantages and it is difficult to determine the accurate method to evaluate antioxidant capacities quantitatively. Previous studies reported correlation between ORAC and TEAC methods, DPPH• and TEAC methods, FRAP and ORAC methods and FRAP and DPPH• methods [50,51]. In the present study, DPPH• assay was found correlated with ORAC assay (*R* = 0.852 *p* ≤ 0.06) and ABTS•+ assay with FRAP assay (*R* = 0.952 *p* ≤ 0.01).

Collectively, all methods indicated that *Agastache* honey has significant antioxidant capacity. The main therapeutic role of antioxidants of honey is to fulfil the requirement of radical scavengers when ROS level is elevated due to microbial infection and inflammation. The supply of antioxidants is necessary to reduce the risk of infectious diseases and improve the immune function. Moreover, the phenolic compounds and the percentage of individual compounds in honey are responsible for its antioxidant activity. It is also important to note that most of the honeys which have been demonstrated to possess higher antioxidants have not been considered for therapeutic applications and their efficacy in medicinal use is due to their antimicrobial activity. Therefore, *Agastache* honey can be considered for the medical applications. However, the antimicrobial activity and the phenolic compounds composition analysis of the *Agastache* honey should be taken into consideration for the future work.

## 3. Materials and Methods

### 3.1. Mono-floral Agastache Honey Production

*Agastache rugosa* was grown in a sealed PC-2 glasshouse facility during March–August (2015) under controlled conditions. Glasshouse was equipped with sensors where temperature and moisture content in the air was logged automatically every day. Two hundred and fifty plants were grown up to flower stage and transferred to another sealed greenhouse where bee-hive was placed. All samples were collected aseptically from the shaped combs. Sampling was carried out during the months of November to February on sunny days at room temperature (25–35 °C). Honey samples in sterile tubes were stored at 4 °C and processed within 24–48 h after harvest time. Other commercial honey samples such as Manuka honey (hnz, UMF 22+, *Leptospermum scoparium*), Tea-tree honey (Miellerie, *Leptospermum lanigerum & Leptospermum scoparium*), Jelly bush honey (Australia’s Manuka, 20+ Active, *Leptospermum polygalifolium*), Super manuka honey (Berringa, MGO-400, *Leptospermum polygalifolium*) and Jarrah honey (Elixir, TA 45+ *Eucalyptus marginata*) were purchased from the local store.

### 3.2. Physico-Chemical Properties

#### 3.2.1. pH Measurement

A pH meter (TPS, 900-P, Brisbane, Australia) was used to measure the pH of a 10% (*w*/*v*) solution of honey prepared in Milli-Q water (Millipore Corporation, Melbourne, Australia).

#### 3.2.2. Moisture Content

The refractive indices of the honey samples were measured at ambient temperature using a handheld refractometer (SHIBUYA, 22878, Saitama, Japan). The readings were further corrected for a standard temperature of 20 °C by adding a correction factor of 0.00023/°C. The moisture content was measured in triplicate and the percentage of moisture content corresponding to the corrected refractive index was calculated using Wedmore’s table (AOAC, 1990) [52].

#### 3.2.3. Determination of Protein Content

The protein content was determined by Bradford’s method [53]. To 10 µL of honey solution (50% *w*/*v*), 500 µL of the Coomassie Brilliant Blue reagent (200 mg of Coomassie Brilliant Blue G-250 dissolved in 100 mL 95% ethanol and 200 mL 85% phosphoric acid (H_3_PO_4_), the resulting solution diluted to a final volume of 2 L) was added. The reagent forms a blue complex with the protein. After 5 min of incubation, the absorbance was measured at 595 nm against the blank (without the sample) using a spectrophotometer (FLUOstar Omega, BMG Labtech, Melbourne, Australia). Bovine serum albumin (BSA) was used for the calibration curve (10–100 µg/mL) in 0.15 M sodium chloride. The protein content was calculated and expressed as µg/g of BSA/g of honey.

#### 3.2.4. Colour Analysis

The colour characteristic of honey was determined using the method described by Beretta et al. [15]. Honey samples were heated to 50 °C to dissolve sugar crystals and the colour was determined by spectrophotometric measurement of the absorbance of a 50% honey solution (*w*/*v*) at 450 nm and 720 nm. The difference in the absorbance readings was expressed in mAU.

#### 3.2.5. Determination of Total Phenolic Content

The Folin-Ciocalteu method was used to determine total phenolic compounds [54]. Honey was dissolved in distilled water to the concentration of 1 g/mL, mixed and filtered by using qualitative filters (Whatman, a No. 40 filter paper). The volume of 200 µL was mixed with 1000 µL of FC reagent previously diluted with the distilled water in proportion 1:10. After 6 min standing in the dark, 800 µL of saturated sodium carbonate solution was added, shaken and left in the dark for 2 h to react. After reaction, absorbance was recorded at 740 nm. The total phenolic content was expressed as µg of Gallic Acid Equivalent (GAE) per gram of honey as the concentration of honey was 1g/mL.

#### 3.2.6. Determination of Flavonoid Content

Flavonoid content of the honey was determined by the procedure described by Barros et al. [55]. Sample (250 µL) was mixed with 1.25 mL of distilled water and 75 µL of a 5% sodium nitrite solution. After 5 min, 150 µL of a 10% aluminium chloride solution was added. After 6 min, 500 µL of 1 M sodium hydroxide and 275 µL of distilled water were added to the mixture. The intensity of the pink colour solution was measured at 510 nm. (+)-Catechin was used as a reference standard to calculate the standard curve and the results were expressed as µg of Catechin Equivalent (CE) per g of honey.

### 3.3. Antioxidant Capacity

#### 3.3.1. Determination of Radical Scavenging Capacity against DPPH• (Antiradical Activity)

The antioxidant capacity of honey samples was assessed by DPPH• assay according to procedure described by Sanchez-Moreno et al. [56]. All honeys were dissolved in distilled water to obtain the concentration of 1 g/mL. Volumes of 200 µL of honey solution were mixed with 1800 µL of methanol solution of DPPH• (0.04 mg/mL) and left to stand to react in the dark at room temperature for 30 min. After incubation, the absorbance was read at 517 nm. The DPPH• scavenging activity was calculated using following equation:

DPPH• scavenging activity (%) = [1 − Ax/A_0_] × 100

where A_0_ is the absorbance obtained by using distilled water and Ax is the absorbance of remaining DPPH• after reaction with trolox and honey solution. A calibration curve was constructed for % inhibition by trolox. Results were expressed as micromoles of trolox equivalents per gram of honey (µmol TE/g of honey).

#### 3.3.2. Free Radical Scavenging Capacity against ABTS•+ (TEAC Assay)

The free radical scavenging activity was determined according to the method of Baltrusaityte et al. [57] with some modifications reported by Brangoulo et al. [58]. The ABTS•+ free radical solution was prepared (final concentration-7 mM/L) with potassium persulphate (final concentration-2.45 mM/L) and left to react for 16 h to form the stable ABTS•+ radical cation. The ABTS•+ solution was further diluted (5 times) to get absorbance between 2–2.4 at 645 nm. A standard of trolox solution was prepared in the range of 2–0.125 mmol/L. The honey samples were prepared in water at 1 g/mL. A 96-well flat-bottomed plate was loaded with 100 µL of sample and 100 µL of ABTS•+ solution was injected into each well using FLUOstar Omega microtiter plate reader (BMG LabTech, Australia) operated at 25 °C. The absorbance of the sample and trolox was read before injecting ABTS•+ solution and after injecting sample to obtain corrected values. The free-radical scavenging antioxidant activity was expressed as trolox-equivalent antioxidant capacity (TEAC), in µmol of trolox per g of honey calculated according to equation:

ABTS•+ scavenging activity (%) = [1 − Ax/A_0_] × 100

#### 3.3.3. Oxygen Radical Absorbance Capacity Assay

The ORAC assay was based on the procedure described by Gillespie et al. [59]. In this assay, free radicals are produced by AAPH and the fluorescein is oxidized, losing its fluorescence. All reagents were prepared in phosphate buffer (pH 7.0) and trolox (5 µg/mL, 20 µM, final concentration) was used as standard. Each well of the plate reader contained in the final volume of 200 µL assay solutions constituted by: fluorescein (16.7 nM), 1–10 mg/mL of honey and AAPH 2.2 mg/mL (final concentration). After addition of AAPH the plate was shaken for 5 s, then the fluorescence was measured every 60 s for 110 cycles with emission and excitation wavelength of λ = 535 and 485 nm. All fluorescence measurements were made at 37 °C and the ORAC values were calculated as area under the curve (AUC) and expressed as µmol trolox equivalent (TE)/g. A blank contained AAPH, fluorescein and phosphate buffer (pH 7) was included.

#### 3.3.4. Ferric Reducing Antioxidant Power Assay

The FRAP assay was carried out as previously described by Benzie et al. [60] with some minor modifications [51]. The FRAP reagent contained 2.5 mL of a 10 mM TPTZ (2,4,6-tripyridyl-s-triazine) solution in 40 mM HCl, 2.5 mL of 20 mM FeCl_3_ and 25 mL of 0.3 M acetate buffer, pH 3.6. It was prepared daily and kept in the dark at 37 °C. Aliquots of 200 µL of honey solution were mixed with 1.8 mL of FRAP reagent and the absorbance of the reaction mixture was measured spectrophotometrically at 593 nm after incubation at 37 °C for 10 min. Trolox (0–500 µM) was used for the calibration curves and the results were expressed as µmoles of trolox equivalents per g of honey (µmol TE/g of honey).

### 3.4. Statistical Analyses

All assays were performed in triplicates and the results were expressed as mean values of three determinations with standard error (SE). Correlations were established using Pearson’s correlation coefficient (*R*) and *p* < 0.05 were regarded as significant. These correlations were calculated using statistical software, Minitab 16 and Microsoft office Excel 2007.

## Figures and Tables

**Table 1:** Physico-chemical analysis of six mono-floral honeys (means ± standard errors).

Honey Samples	pH	Moisture (%)	Protein (µg/g)	Colour (A_450_, mAU)
*Agastache*	4.10 ± 0.1	17.0 ± 0.5	1428 ± 83.4	461 ± 8.8
Manuka	4.03 ± 0.2	19.5 ± 0.3	903 ± 107	1007 ± 4.0
Tea tree	4.10 ± 0.1	20.0 ± 0.15	1319 ±18.4	507 ± 15.4
Jelly bush	3.84 ± 0.23	14.5 ± 0.5	1384 ± 64.2	1135 ± 3.1
Super manuka	3.83 ± 0.12	17.0 ± 0.35	1016 ± 143.1	726 ± 3.7
Jarrah	4.30 ± 0.12	21.0 ± 0.5	2178 ± 100	518 ± 2.6

**Table 2:** Phenolic content, flavonoid content and antioxidant capacities of honeys (means ± standard error).

Honey Samples	TPC (GAE µg/g)	TFC (CE µg/g)	DPPH• (µmol TE/g)	TEAC (µmol TE/g)	ORAC (µmol TE/g)	FRAP (µmol TE/g)
*Agastache*	853.6 ± 5.0	26.67 ± 5.6	9.85 ± 1.98	26.88 ± 0.32	19.78 ± 1.1	3.61 ± 0.02
Manuka	1288.0 ± 102.8	37.64 ± 7.2	18.69 ± 0.9	30.72 ± 0.27	24.82 ± 0.5	3.68 ± 0.04
Tea-tree	1263.5 ± 143.1	20.08 ± 4.3	17.25 ± 1.7	13.60 ± 0.35	14.16 ± 0.2	2.72 ± 0.16
Jelly bush	1415.6 ± 126	53.91 ± 10.9	17.25 ± 0.8	23.84 ± 0.29	26.95 ± 0.9	3.36 ± 0.15
Super manuka	974.4 ± 26.9	24.90 ± 4.3	11.34 ± 0.69	21.28 ± 0.14	12.40 ± 0.3	3.28 ± 0.02
Jarrah	1028.7 ± 27.4	39.3 ± 8.9	6.87 ± 0.77	20.96 ± 0.33	12.44 ± 0.5	3.34 ± 0.03

**Table 3:** Correlation matrix showing the correlations between the colour, TPC, TFC and DPPH• scavenging capacity.

	COLOUR	TPC	TFC	DPPH•
DPPH•	0.925	0.826	0.484	
TFC	0.685	0.866		
TPC	0.944			
COLOUR				
